# Fructose Diet–Induced Liver Injury Through Oxidative Stress: A Systematic Review of Preclinical Studies

**DOI:** 10.1155/jnme/1644860

**Published:** 2026-05-07

**Authors:** Marissa Arifin, Wardatul Jannah, Neily Zakiyah, Anna Meiliana, Melisa Intan Barliana, Keri Lestari

**Affiliations:** ^1^ Doctoral Program in Pharmacy, Faculty of Pharmacy, Universitas Padjadjaran, Sumedang, 45363, West Java, Indonesia, unpad.ac.id; ^2^ Department of Pharmacology and Clinical Pharmacy, Faculty of Pharmacy, Universitas Padjadjaran, Sumedang, 45363, West Java, Indonesia, unpad.ac.id; ^3^ Prodia Clinical Laboratory, Central Jakarta 10430, Jakarta, Indonesia; ^4^ Drug Utilization and Pharmacoepidemiology Research Group, Center of Excellence for Pharmaceutical Care Innovation, Universitas Padjadjaran, Sumedang, 45363, West Java, Indonesia, unpad.ac.id; ^5^ Centre of Excellence for Pharmaceutical Care Innovation, Universitas Padjadjaran, Sumedang, 45363, West Java, Indonesia, unpad.ac.id; ^6^ Prodia Education and Research Institute, Central Jakarta 10430, Jakarta, Indonesia; ^7^ Department of Biological Pharmacy, Faculty of Pharmacy, Universitas Padjadjaran, Sumedang, 45363, West Java, Indonesia, unpad.ac.id

**Keywords:** animal study, fructose diet, hepatic oxidative stress, oxidative stress, pre-clinical trial

## Abstract

**Background:**

Fructose consumption has significantly increased in recent years and is associated with hepatic oxidative stress, playing a major role in metabolic diseases such as metabolic‐associated fatty liver disease (MAFLD). This systematic review aimed to summarize how excessive fructose consumption causes liver injury through oxidative stress, leading to lipid accumulation in hepatic cells.

**Methods:**

PubMed, Scopus, and Web of Science databases were systematically searched (2019–2024) for preclinical studies using fructose‐only diets. Criteria were applied to identify relevant studies. The primary outcome was hepatic oxidative stress, and the secondary outcomes included weight, metabolic markers, liver function, and histopathology changes. Two reviewers assessed bias risk using SYRCLE.

**Results:**

Among 517 studies screened, 26 met the inclusion criteria. Most studies had unclear bias risk due to poor reporting. Low‐dose fructose intake (10%) over 8–12 weeks induced hepatic oxidative stress, indicated by elevated malondialdehyde (MDA) in 53.8% of cases, increased reactive oxygen species (ROS) in 19.2%, and reduced antioxidant defenses, including glutathione (GSH), GSH peroxidase (GSH‐Px), and superoxide dismutase (SOD) in 38.5%. Four studies showed early upregulation of antioxidant responses (11.5%), suggesting initial adaptation. Prolonged fructose exposure (up to 36 weeks) caused sustained liver injury due to overwhelming defenses and increased oxidative stress. Most studies also reported metabolic disturbances, liver dysfunction, and lipid accumulation.

**Conclusion:**

This systematic review showed that excessive fructose consumption induces liver injury through oxidative stress, which then triggers subsequent processes like inflammation. Overconsumption led to uncontrolled hepatic metabolism, increasing lipid synthesis, metabolic overload, overproduction of ROS, impairment of antioxidant defenses, and histopathological changes.

## 1. Introduction

The change from nonalcoholic fatty liver disease (NAFLD) to metabolic‐associated fatty liver disease (MAFLD) nomenclature, proposed by the Asian Pacific Association for the Study of the Liver (APASL) in 2020, is positively impacting disease management policies [1–3]. The impact has increased fresh enthusiasm among those focused on studying and addressing the condition [3]. This update introduces MAFLD as a promising new concept that enhances the comprehension, management, and investigation of fatty liver disease linked to metabolic dysfunction [1]. The change also aims to facilitate study progress to discover novel and more effective therapies for chronic liver diseases, thereby improving patient outcomes [4].

The key diagnostic difference between NAFLD and MAFLD is in the procedural methods. NAFLD uses an exclusion‐based method, requiring evidence of liver fat without significant alcohol use or other liver diseases. In comparison, MAFLD adopts an inclusion‐based method, diagnosed by liver fat accumulation or steatosis alongside metabolic conditions such as obesity, diabetes, or other metabolic risk factors, regardless of alcohol intake or other liver conditions [3]. MAFLD is diagnosed when there is an indication of hepatic steatosis accompanied by at least one of these three factors: overweight or obesity, type 2 diabetes mellitus, or metabolic dysfunction in individuals of lean body weight [1].

Unhealthy dietary habits, particularly increased consumption of fructose‐containing sweeteners, have been strongly associated with the development of metabolic disorders and liver dysfunction [5]. The widespread use of high‐fructose corn syrup (HFCS) and other fructose‐rich additives in processed foods has substantially increased daily fructose intake, contributing to metabolic dysregulation and hepatic lipid accumulation [6, 7]. Among dietary factors, high fructose consumption has become a significant concern due to the harmful health impacts [6]. Excessive fructose consumption, particularly from high‐fructose diets, has been related to the development of fat accumulation and liver dysfunction. In liver, fructose metabolism bypasses essential regulatory steps of glycolysis, leading to increased lipogenesis, insulin resistance, and oxidative stress [8, 9]. In particular, hepatic oxidative stress significantly contributes to the progression of liver injury by promoting inflammation, mitochondrial dysfunction, and fibrosis, causing a progression of the MAFLD [8, 9]. To better understand this mechanism, animal models are often used to explore the biochemical and molecular pathways behind diet‐induced liver injury.

Many studies have examined how high‐fructose diet impacts oxidative stress markers, antioxidant defenses, and associated pathways. However, the results vary significantly, attributed to differences in animal species, fructose concentration, and duration of follow‐up. This shows the need for a systematic review to synthesize the existing results and comprehensively assess the effect of a fructose diet on hepatic oxidative stress. Therefore, this systematic review aimed to analyze the impact of fructose‐induced hepatic oxidative stress. The results were expected to contribute to a better understanding of fructose‐induced hepatic dysfunction and implications for human health, particularly concerning MAFLD and metabolic diseases.

## 2. Materials and Methods

### 2.1. Registration of Protocol

This systematic review was conducted following the Preferred Reporting Items for Systematic Reviews and Meta‐Analyses (PRISMA) checklist (see Supporting Information 1) [10]. The protocol was registered with the International Prospective Register of Systematic Reviews (PROSPERO) 2025 (https://www.crd.york.ac.uk/PROSPERO/view/CRD420251009741). The review title was amended after PROSPERO registration to better reflect the refined focus of the study. No other changes were made to the registered protocol (see Supporting Information 2) [11].

### 2.2. Search Strategy and Eligibility Criteria

The literature was systematically searched using three databases, namely, MEDLINE (PubMed), Scopus, and Web of Science, and adjusted with medical subject headings (MeSH) terms. The search terms used as keywords were “high fructose diet” OR “fructose diet” AND “hepatic oxidative stress.” Details of the search strategy are provided in Supporting Information 3, Table S1.

The studies included were selected using the Population, Intervention, Comparison, and Outcome (PICO) framework. The selected population focused exclusively on animal models (all species and all sexes) limited to studies using fructose diet as the intervention, exclusively without combining with another diet. This systematic review included studies published in English over the past five years (2019–2024). The comparator was the control group with regular diets without fructose. Analyzing the impact of fructose‐induced hepatic oxidative stress included examining two outcomes. First, as the primary outcome, hepatic oxidative stress was assessed using several biomarkers, including malondialdehyde (MDA), glutathione (GSH), GSH peroxidase (GSH‐Px), superoxide dismutase (SOD), and other related oxidative stress. Second, as a secondary outcome, changes in weight, metabolic status, and liver histopathology were evaluated.

This systematic review excluded studies not conducted using in vivo animal models. Other exclusion criteria were studies without a control group, lack of primary and secondary outcomes, reviews, conference papers, and proceedings. Studies that could not be accessed in full text after requesting access from the author were also excluded.

### 2.3. Study Selection

The search records from all databases were exported to Rayyan.ai.new [12], a web‐based intelligent systematic review application and checked for duplicates. Screening processes were conducted in two stages. The initial screening was based on the title and abstract, followed by full‐text screening. Two authors (MA and WJ) reviewed the stages independently. Any discrepancies were resolved by consensus or discussions with a third and fourth author (NZ and AM).

### 2.4. Data Extraction and Synthesis

A data extraction sheet was developed to collect essential characteristics and information for eligible studies, including the author’s name, year of publication, country of study, population species, sex, and sample size. Furthermore, details regarding the intervention strategy were documented, including fructose concentration, duration of follow‐up, and the study outcomes.

This systematic review used a narrative synthesis to summarize and interpret the results across the included studies. The decision to use narrative synthesis was based on the heterogeneity in study designs, outcome measures, and methods, which limited the feasibility of a quantitative meta‐analysis. This method allowed for a structured and transparent synthesis of results, showing patterns, relationships, and differences among studies. Data were organized thematically, considering study quality, population characteristics, and intervention effects to ensure a comprehensive and coherent interpretation of the results.

### 2.5. Quality Assessment

Two authors (MA and WJ) independently assessed the risk of bias using the Systematic Review Center for Laboratory Animal Experimentation (SYRCLE) (SYRCLE’s RoB tool) [13, 14]. Identifying six types of bias, namely, selection bias, performance bias, detection bias, attrition bias, reporting bias, and other biases, each domain was classified into three levels, namely, high, low, or unclear risk [13, 14]. Any discrepancies were resolved by consensus or discussions with two authors (NZ and AM). We generated traffic‐light and summary plots using the risk‐of‐bias visualization (robvis) tool to summarize the risk of bias [15].

## 3. Results

### 3.1. Study Selection

**FIGURE 1:**
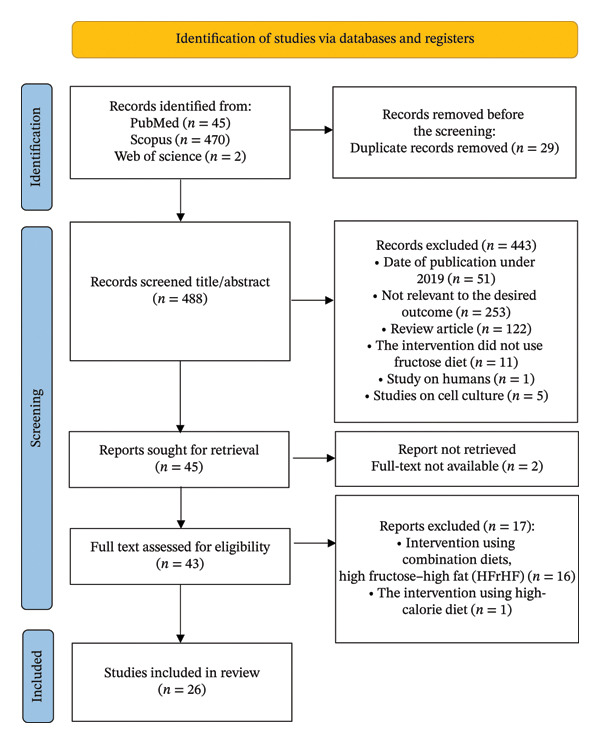
PRISMA flow diagram. PRISMA, Preferred Reporting Items for Systematic Reviews and Meta‐analyses.

A total of 517 records were sourced from PubMed, Scopus, and Web of Science. Twenty‐six duplicates were removed using Rayyan.ai [12]. After applying exclusion criteria, 443 were excluded during the screening title and abstract. A total of 17 records were excluded during full‐text screening because of using a combination diet (high‐fructose–high‐fat diet) and a high‐calorie diet. A total of two had unavailable full texts. Only 26 studies were included in the review [16–41]. The study selection process is illustrated in the PRISMA flow diagram (Figure 1).

### 3.2. Characteristics of the Included Studies

Table 1 presents the general characteristics of the 26 preclinical studies that meet the inclusion criteria [16–41]. All studies were conducted using rats or mice (26/26, 100%) [16–41], and the sex of the animals used in the experiments was reported in most cases (25/26, 96.2%) [16–38, 40, 41]. Although our search strategy and eligibility criteria did not restrict inclusion based on the sex of the animal models, we observed that the included studies predominantly used male animals (24/26, 92.3%), while one study exclusively used female (1/26, 3.8%) [26]. One study did not report the sex of the animals used (1/26, 3.8%) [39]. This reflects a common practice in preclinical research to use male subjects to avoid confounding hormonal variations from female models [42]. While not a result of intentional bias in our methodology, it introduces a limitation in terms of generalizability, particularly regarding sex‐specific difference in fructose metabolism and hepatic oxidative stress.

**TABLE 1:** Characteristics of included studies (*n* = 26).

No. of study	Author	Country	Population species	Sex	Sample size	Control	Control group	Intervention group	Form of fructose administered	Concentration of fructose (%)	Follow‐up (week)
*Fructose 10%*											
1	Règgami et al. [33]	Algeria	Rats	Male	42	Yes	Normal control (NC)	Fructose‐drinking (FC)	Solution	10	9
2	Alemán, et al. [17]	Argentina	Rats	Male	24	Yes	Control	High‐fructose	Solution	10	12
3	Yang et al., [39]	Germany	Mice	N/A	80	Yes	Control	Fructose‐fed	Solution	10	12
4	Abdelhamid et al. [16]	Egypt	Rats	Male	30	Yes	Control	Metabolic syndrome (MS)	Solution	10	16
5	Zouaoui et al. [41]	England	Rats	Male	56	Yes	Control	High‐fructose diet (HFD)	Solution	10	20

*Fructose 20%*											
6	Wang et al. [37]	China	Mice	Male	40	Yes	Normal	High fructose (HF)	Solution	20	8
7	Gubur et al. [24]	Turkey	Rats	Male	32	Yes	Control (C)	Fructose (F)	Solution	20	8
8	Alim et al. [18]	China	Rats	Male	40	Yes	Normal diet (ND)	High fructose–fed (HF)	Solution	20	10
9	Altintas et al. [19]	Turkey	Rats	Male	39	Yes	Control	High‐fructose diet (FED)	Solution	20	16
10	Hernández et al. [25]	Mexico	Rats	Male	30	Yes	Control	Fructose‐fed	Solution	20	18
11	Mautone Gomes et al. [28]	United States	Rats	Male	24	Yes	Control	Fructose	Solution	20	24

*Fructose 30%*											
12	Bingül et al. [21]	Turkey	Rats	Male	34	Yes	Control	High‐fructose diet (HFr)	Solution	30	8
13	Bingul et al. [20]	Turkey	Rats	Male	40	Yes	Control	High‐fructose (HFr)	Solution	30	8
14	EL Shial et al. [23]	Egypt	Mice	Male	54	Yes	Normal control (NC)	High‐fructose diet (HFD)	Solution	30	8
15	Li et al. [38]	China	Mice	Male	32	Yes	Control	High‐fructose	Solution	30	8
16	Iskender et al. [27]	Turkey	Rats	Male	24	Yes	Control (W)	Fructose	Solution	30	12
17	Zhang et al. [40]	England	Mice	Male	30	Yes	Normal diet (ND)	High‐fructose diet (HFD)	Solution	30	13
18	Quan et al. [30]	United States	Mice	Male	40	Yes	Normal control (Con)	High fructose	Solution	30	16

*Fructose ≥ 40%–60%*											
19	Hsu et al. [26]	Taiwan	Mice	Female	32	Yes	Control	High‐fructose diet (HFD)	Solid	44	8
20	Sakamuri et al. [34]	Scotland	Rats	Male	48	Yes	Control	High‐fructose diet (FR‐215)	Solid	54	36
21	Elseweidy et al. [22]	Egypt	Rats	Male	24	Yes	Normal control	High‐fructose diet (HFD)	Solid	60	8
22	Shawky NM et al. [35]	Netherlands	Rats	Male	34	Yes	Control	High‐fructose diet (HFrD)	Solid	60	9
23	Park et al. [29]	Korea	Rats	Male	30	Yes	N/A	High‐fructose diet (HFD)	Solution	60	10
24	Singh et al. [36]	India	Rats	Male	24	Yes	Control	High‐fructose diet (HFrD)	not mention	60	10
25	Rashwan et al. [32]	Egypt	Rats	Male	50	Yes	Control	Fructose‐fed group	Solid	60	14

*Fructose > 60%*											
26	Rai et al. [31]	India	Rats	Male	30	Yes	Control	High‐fructose‐fed	Solid	65	8

A total of 5 studies were conducted in Europe (England, Scotland, Germany, and Netherlands) [34, 35, 39–41], 12 studies in Asia (China, India, Korea, Taiwan, and Turkey) [18–21, 24, 26, 27, 29, 31, 36–38], 3 studies in North America (United States and Mexico) [25, 28, 30], 1 study in South America (Argentina) [17], and 5 studies in Africa (Egypt and Algeria) [16, 22, 23, 32, 33].

The protocol for fructose diet concentration varied among these preclinical studies, ranging from 10% to 65%. Fructose was present in solutions in drinking water and in solid or pellet form, administered orally. Additionally, one study did not specify the form of fructose that was administered [36]. A total of 19 studies used fructose in solution form in drinking water (19/26, 73.1%) [16–21, 23–25, 27–30, 33, 37–41], and six were conducted with solid or pellet form (6/26, 23.1%) [22, 26, 31, 32, 34, 35]. Based on search, five studies used a 10% fructose diet (5/26, 19.2%) [16, 17, 33, 39, 41], six used a 20% fructose diet (6/26, 23.1%) [18, 19, 24, 25, 28, 37], and seven applied a 30% fructose diet (7/26, 26.9%) [20, 21, 23, 27, 30, 38, 40]. Meanwhile, one used a 44% fructose diet (1/26, 3.8%) [26], 54.4% fructose diet (1/26, 3.8%) [34], and 65% fructose diet (1/26, 3.8%) [31], respectively, and five applied a 60% fructose diet (5/26, 19.2%) [22, 29, 32, 35, 36]. Overall, most studies used low to moderate fructose concentrations (10%–30%) (18/26, 69.2%) [16–21, 23–25, 27, 28, 30, 33, 37–41], whereas very high fructose doses (≥ 44%–60%, and > 60%) were examined in a limited number of experimental models (8/26, 30.8%) [22, 26, 29, 31, 32, 34–36].

The duration of the intervention ranged from 8 to 36 weeks. Among the 26 studies, the most frequently used intervention duration was 8 weeks (9/26, 34.6%) [20–24, 26, 31, 37, 38], followed by 10 weeks (3/26, 11.5%) [18, 29, 36], 12 weeks (3/26, 11.5%) [17, 27, 39], and 16 weeks (3/26, 11.5%) [16, 19, 30]. The remaining eight studies had intervention durations of 8.5, 9, 13, 14, 18, 20, 24, and 36 weeks (1/26, 3.8%) [25, 28, 32–35, 40, 41].

### 3.3. Outcomes Study

Table 2 presents the main findings of the measured outcomes. Among the 26 studies, only one did not report any primary outcomes (1/26, 3.85%) [26], while the secondary outcome was reported in all [16–41]. Table 3 and Supporting Information Table S2 present the outcome biomarkers of the studies included. A total of nine studies reported three biomarkers (MDA, GSH/GSH‐Px, and SOD) (9/26, 34.6%) [17, 18, 24, 30, 34, 35, 37, 39, 40]. In line with the search, four studies did not measure specific hepatic oxidative stress biomarkers but assessed others (4/26, 15.4%) [21, 23, 28, 29]. Meanwhile, 12 studies measured only one or two biomarkers (12/26, 46.2%) [16, 19, 20, 22, 25, 27, 31–33, 36, 38, 41]. Notably, the primary outcomes were assessed exclusively in male animal models [16–25, 27–41], with no study evaluating oxidative stress biomarkers in female models [26], highlighting a significant sex bias in the investigation of hepatic stress oxidation. In contrast, the secondary outcomes were consistently observed across both male and female animal models [16–41]. These included significant increases in body weight, alterations in metabolic parameters such as insulin resistance and lipid profiles, elevated serum liver enzyme levels (e.g., ALT, AST, and ALP) indicative of hepatic injury, and notable histopathological changes.

**TABLE 2:** Main findings of the outcomes measured.

No. of study	Author	Concentration of fructose (%)	Duration of follow‐up (weeks)	Form of fructose administered	Outcomes					
					Primary outcomes			Secondary outcomes		
					Induced hepatic oxidative stress	Induced antioxidant biomarkers	Induced weight changes	Induced metabolic changes	Increased liver enzyme biomarkers	Induced histopathological changes
*Fructose 10%*										
1	Règgami et al. [33]	10	9	Solution	⬆^∗^	⬇^∗^	⬆	⬆^∗^	X	Yes
2	Alemán et al. [17]	10	12	Solution	⬆^∗^	⬆^∗^	⬆^∗^	⬆^∗^	⬆^∗^	Yes
3	Yang et al. [39]	10	12	Solution	⬆^∗^	⬇^∗^	⬆^∗^	⬆^∗^	⬆^ᴼ^	Yes
4	Abdelhamid et al. [16]	10	16	Solution	⬆^∗^	X	⬆^∗^	⬆^∗^	⬆^∗^	Yes
5	Zouaoui et al. [41]	10	20	Solution	X	⬇^ᴼ^	X	⬆^ᴼ^	⬆^ᴼ^	Yes

*Fructose 20%*										
6	Wang et al. [37]	20	8	Solution	⬆^∗^	⬇^∗^	⬆^ᴼ^	⬆^∗^	⬆^∗^	X
7	Gubur et al. [24]	20	8	Solution	⬆^ᴼ^	⬆^ᴼ^	⬆	⬆^∗^	⬆^∗^	Yes
8	Alim et al. [18]	20	10	Solution	⬆^∗^	⬇^ᴼ^	⬆^ᴼ^	⬆^∗^	⬆^∗^	Yes
9	Altintas et al. [19]	20	16	Solution	⬆^∗∗^	⬆^∗∗^	X	X	X	Yes
10	Hernández et al. [25]	20	18	Solution	X	⬇^∗^	⬆^∗^	⬆^∗^	⬆^∗^	Yes
11	Mautone Gomes et al. [28]	20	24	Solution	⬆^∗^	X	⬆	⬆^∗^	⬆^∗^	X

*Fructose 30%*										
12	Bingül et al. [21]	30	8	Solution	⬆^∗^	X	X	⬆^∗^	⬆^∗^	Yes
13	Bingul et al. [20]	30	8	Solution	X	⬇^∗^	⬆	⬆^∗^	⬆^∗^	Yes
14	EL Shial et al. [23]	30	8	Solution	⬆^∗^	X	X	⬆^∗^	⬆^∗^	Yes
15	Li et al. [38]	30	8	Solution	X	⬇^∗^	⬆^ᴼ^	⬆^∗^	⬆^∗^	X
16	Iskender et al. [27]	30	12	Solution	⬆^∗^	⬇^∗^	X	⬆^∗^	X	Yes
17	Zhang et al. [40]	30	13	Solution	⬆^∗^	⬇^∗^	⬆^ᴼ^	⬆^∗^	⬆^∗^	Yes
18	Quan et al. [30]	30	16	Solution	⬆^∗^	⬇^∗^	⬆^∗^	⬆^∗^	X	Yes

*Fructose ≥ 40%–60%*										
19	Hsu et al. [26]	44	8	Solid	X	X	⬆^∗^	⬆^∗^	⬆^∗^	Yes
20	Sakamuri et al. [34]	54	36	Solid	⬆^ᴼ^	⬇^ᴼ^	⬆^ᴼ^	⬆^ᴼ^	X	Yes
21	Elseweidy et al. [22]	60	8	Solid	⬆^∗^	⬆^∗^	⬆^∗^	⬆^∗^	⬆^∗^	Yes
22	Shawky et al. [35]	60	9	Solid	⬆^∗^	⬆^∗^	X	⬆^∗^	X	Yes
23	Park et al. [29]	60	10	not mention	X	⬇^∗^	X	⬆^ᴼ^	⬆^ᴼ^	Yes
24	Singh et al. [36]	60	10	Solution	⬆^ᴼ^	X	⬆	⬆^∗^	⬆^∗^	Yes
25	Rashwan et al. [32]	60	14	Solid	X	⬇^∗^	⬇^∗^	⬆^∗^	⬆^∗^	X

*Fructose > 60%*										
26	Rai et al. [31]	65	8	Solid	X	⬇^∗∗^	⬆^ᴼ^	⬆^∗^	X	Yes

*Note:* ⬆, increase; ⬇, decrease; X, not analyzed.

^∗^significant with *p* value ≤ 0.05.

^∗∗^Not significant with *p* ≥ 0.05.

^ᴼ^ *p* value not reported.

**TABLE 3:** Summary of the outcome measured.

Category	Finding measure(s)	Specifics	Number (%)
Primary outcomes	Three biomarkers of oxidative stress (MDA, GSH or GSH‐Px, and SOD	Reported all of three biomarkers (MDA, GSH or GSH‐Px, and SOD) with others stress oxidative biomarkers	4/26 (15.4)
		Reported three biomarkers (MDA, GSH or GSH‐Px, and SOD) only without others stress oxidative biomarker	5/26 (19.2)
		Reported one or two of three biomarkers (MDA, GSH or GSH‐Px, and SOD) and other biomarkers	12/26 (46.2)
		Reported other biomarkers	4/26 (15.4)
		Not reported	1/26 (3.8)
	MDA	Reported	14/26 (53.8)
		Significantly increased^∗^	11/26 (42.3)
		Significantly decreased^∗^	—
		Not significant	1/26 (3.8)
		Did not report *p* value (increase/decrease)	2/26 (7.7)
		Not reported	12/26 (46.2)
	GSH/GSH‐Px	Reported	17/26 (65.4)
		Significantly increased^∗^	4/26 (11.5)
		Significantly decreased^∗^	10/26 (38.5)
		Not significant	
		Did not report *p* value (increase/decrease)	4/26 (15.4)
		Not reported	9/26 (34.6)
	SOD	Reported	18/26 (69.2)
		Significantly increased^∗^	3/26 (11.5)
		Significantly decreased^∗^	10/26 (38.5)
		Not significant	1/26 (3.8)
		Did not report *p* value (increase/decrease)	4/26 (15.4)
		Not reported	8/26 (30.8)
	Other biomarkers	Reported	11/26 (42.3)
		Significantly increased^∗^	7/26 (26.9)
		Significantly decreased^∗^	—
		Not significant	1/26 (3.8)
		Did not report *p* value (increase/decrease)	3/26 (11.5)
		Not reported	15/26 (57.7)

Secondary outcomes	Weight changes	Reported	19/26 (73.1)
		Significantly increased^∗^	7/26 (26.9)
		Significantly decreased^∗^	1/26 (3.8)
		Not significant	6/26 (23.1)
		Did not report *p* value (increase/decrease)	5/26 (19.2)
		Not reported	7/26 (26.9)
	Metabolic changes	Reported	25/26 (96.2)
		Significantly increased^∗^	20/26 (76.9)
		Significantly decreased for HDL‐C^∗^	12/26 (46.2)
		Significant decreased for other biomarkers (Apo‐A1, TG, HOMA‐β index)^∗^	3/26 (11.5)
		Did not report *p* value (increase/decrease)	5/26 (19.2)
		Not reported	1/26 (3.8)
	Liver enzyme levels	Reported	19/26 (73.1)
		Significantly increased^∗^	16/26 (61.5)
		Significantly decreased^∗^	—
		Not significant	—
		Did not report *p* value (increase/decrease)	3/26 (11.5)
		Not reported	7/26 (26.9)
	Histopathological changes	Reported	22/26 (84.6)
		Not reported	4/26 (15.4)
		Liver weight	6/26 (23.1)
		Hepatic fat/fatty acid/fat droplet in liver cell/lipid accumulation	11/26 (42.3)
		Steatosis	9/26 (34.6)
		Necrosis of the hepatocytes	5/26 (19.2)
		Inflammation/inflammatory cells	3/26 (11.5)
		Hepatocyte ballooning	5/26 (19.2)

*Note:* Apo‐A1, Apolipoprotein‐A; HDL‐C, high‐density lipoprotein; HOMA‐β, homeostasis model assessment‐β; GSH, glutathione; GSH‐Px, glutathione peroxidase; SOD, superoxide dismutase; MDA, malondialdehyde.

^∗^significances with *p* value ≤ 0.05.

### 3.4. Oxidative Stress in Response to Fructose

Tables 4 and 5 present the observed outcomes by the follow‐up of the intervention (weeks) and fructose dosage (%). All studies included showed outcomes at 10% fructose over a nine‐week follow‐up. The shortest follow‐up was 8 weeks, with doses ranging from 20% to 65%. These dosages were adequate to induce changes in both observed outcomes.

**TABLE 4:** Observed outcomes by follow‐up duration (weeks).

Duration of follow‐up (weeks)	Number of study(s)	Outcomes observed for each duration of follow‐up					
		Induced hepatic oxidative stress	Induced antioxidant	Induced weight changes	Induced metabolic changes	Induced liver enzyme biomarkers	Induced histopathological changes
8	*N* = 9 [20–24, 26, 31, 37, 38]	⬆^∗^ [20–23, 31, 37], ⬆^ᴼ^ [24], X [20, 26, 31, 38]	⬆^∗^ [22], ⬆^ᴼ^ [24], ⬇^∗^ [20, 37, 38], ⬇^∗∗^ [31], X [20, 23, 26]	⬆^∗^ [22, 26], ⬆^ᴼ^ [20, 24, 31, 37, 38], X [21, 23]	⬆^∗^ [20–24, 26, 31, 37, 38]	⬆^∗^ [20–24, 26, 37, 38], X [31]	Yes [20–24, 26, 31], X [37, 38]
9	*N* = 2 [33, 35]	⬆^∗^ [33, 35]	⬆^∗^ [35], ⬇^∗^ [33]	⬆^ᴼ^ [33], X [35]	⬆^∗^ [33, 35]	X [33, 35]	Yes [33, 35]
10	*N* = 3 [18, 29, 36]	⬆^∗^ [18], ⬆^ᴼ^ [29], X [36]	⬇^∗^ [36] ⬇^ᴼ^ [18], X [29]	⬆^ᴼ^ [18, 29], X [36]	⬆^∗^ [18, 29], ⬆^ᴼ^ [36]	⬆^∗^ [18, 29], ⬆^ᴼ^ [36]	Yes [18, 29, 36]
12	*N* = 3 [17, 27, 39]	⬆^∗^ [17, 27, 39]	⬆^∗^ [17], ⬇^∗^ [27, 39]	⬆^∗^ [17, 39], X [27]	⬆^∗^ [17, 27, 39]	⬆^∗^ [17], ⬆^ᴼ^ [39], X [27]	Yes [17, 27, 39]
13	*N* = 1 [40]	⬆^∗^ [40]	⬇^∗^ [40]	⬆^ᴼ^ [40]	⬆^∗^ [40]	⬆^∗^ [40]	Yes [40]
14	*N* = 1 [32]	X [32]	⬇^∗^ [32]	⬆^∗^ [32]	⬆^∗^ [32]	⬆^∗^ [32]	X [32]
16	*N* = 3 [16, 19, 30]	⬆^∗^ [16, 30], ⬆^∗∗^ [19]	⬆^∗∗^ [19], ⬇^∗^ [30], X [16]	⬆^∗^ [16, 30], X [19]	⬆^∗^ [16, 30], X [19]	⬆^∗^ [16], X [19, 30]	Yes [16, 19, 30]
18	*N* = 1 [25]	X [25]	⬇^∗^ [25]	X [25]	⬆^∗^ [25]	⬆^∗^ [25]	Yes [25]
20	*N* = 1 [41]	⬆^ᴼ^ [41]	⬇^ᴼ^ [41]	X [41]	⬆^ᴼ^ [41]	⬆^ᴼ^ [41]	Yes [41]
24	*N* = 1 [28]	⬆^∗^ [28]	X [28]	⬆^∗∗^ [28]	⬆^ᴼ^ [28]	⬆^∗^ [28]	X [28]
36	*N* = 1 [34]	⬆^ᴼ^ [34]	⬇^∗^ [34]	⬆^ᴼ^ [34]	⬆^ᴼ^ [34]	X [34]	Yes [34]

*Note:* ⬆: increase, ⬇: decrease, X: not reported/not analyzed.

^∗^Significant with *p* ≤ 0.05.

^∗∗^Not significant with *p* ≥ 0.05.

^*ᴼ*^Did not provide with significant (*p*) value.

**TABLE 5:** Observed outcomes by concentration of fructose (%).

Concentration of fructose (%)	Number of study(s)	Outcomes observed for each duration of follow‐up					
		Induced hepatic oxidative stress	Induced antioxidant	Induced weight changes	Induced metabolic changes	Induced liver enzyme	Induced histopathological changes
10	*N* = 5 [16, 17, 33, 39, 41]	⬆^∗^ [16, 17, 33, 39], ⬆^ᴼ^ [41]	⬆^∗^ [17], ⬇^∗^ [33, 39], ⬇^ᴼ^ [41], X [16]	⬆^∗^ [16, 17, 39], ⬆^ᴼ^ [33], X [41]	⬆^∗^ [16, 17, 33, 39], ⬆^ᴼ^ [41]	⬆^∗^ [16, 17], ⬆^ᴼ^ [39, 41], X [33]	Yes [16, 17, 33, 39, 41]
20	*N* = 6 [18, 19, 24, 25, 28, 37]	⬆^∗^ [18, 28, 37], ⬆^∗∗^ [19], ⬆^ᴼ^ [24], X [25]	⬆^∗∗^ [19], ⬆^ᴼ^ [24], ⬇^∗^ [25, 37], ⬇^ᴼ^ [18], X [28]	⬆^∗^ [25], ⬆^ᴼ^ [18, 24, 28, 37], X [19]	⬆^∗^ [18, 24, 25, 28, 37], X [19]	⬆^∗^ [18, 24, 25, 28, 37], X [19]	Yes [18, 19, 24, 25], X [28, 37]
30	*N* = 7 [20, 21, 23, 27, 30, 38, 40]	⬆^∗^ [21, 23, 27, 30, 40], ⬆^ᴼ^ [20, 38]	⬇^∗^ [20, 27, 30, 38, 40], X [21, 23]	⬆^∗^ [30], ⬆^ᴼ^ [20, 38, 40], X [21, 23, 27]	⬆^∗^ [20, 21, 23, 27, 30, 38, 40]	⬆^∗^ [20, 21, 23, 38, 40], X [27, 30]	Yes [20, 21, 23, 27, 30, 40], X [38]
44	*N* = 1 [26]	X [26]	X [26]	⬆^∗^ [26]	⬆^∗^ [26]	⬆^∗^ [26]	Yes [26]
54	*N* = 1 [34]	⬆^ᴼ^ [34]	⬇^ᴼ^ [34]	⬆^ᴼ^ [34]	⬆^ᴼ^ [34]	X [34]	Yes [34]
60	*N* = 5 [22, 29, 32, 35, 36]	⬆^∗^ [22, 35], ⬆^ᴼ^ [29], X [32, 36]	⬆^∗^ [22, 35], ⬇^∗^ [32, 36], X [29]	⬆^∗^ [22], ⬆^ᴼ^ [29], ⬇^∗^ [32], X [35, 36]	⬆^∗^ [22, 29, 32, 35], ⬆^ᴼ^ [36]	⬆^∗^ [22, 29, 32], ⬆^ᴼ^ [36], X [35]	Yes [22, 29, 35, 36], X [32]
65	*N* = 1 [31]	X [31]	⬇^∗∗^ [31]	⬆^ᴼ^ [31]	⬆^∗^ [31]	X [31]	Yes [31]

*Note:* ⬆: increase, ⬇: decrease. X: not reported/not analyzed.

^∗^Significant with *p* ≤ 0.05.

^∗∗^Not significant with *p* ≥ 0.05.

^ᴼ^Did not provide with significant (*p*) value.

#### 3.4.1. Oxidative Stress in Response to Fructose Consumption Over Time

Table 4 presents the oxidative stress responses to fructose consumption over time. The duration of fructose intervention plays a crucial role in the onset and progression of oxidative stress. Fructose consumption consistently induced hepatic oxidative stress across all durations studied. Early damage appeared within 8–12 weeks, marked by increased oxidative markers and reduced antioxidant defenses and liver injury [17, 18, 20–24, 26, 27, 29, 31, 33, 35–39]. This stress persisted through 16–20 weeks, indicating sustained oxidative burden [16, 19, 25, 30, 41]. At 24–36 weeks, oxidative markers remained elevated, and antioxidant depletion continued, suggesting worsening stress and impaired recovery with prolonged exposure [28, 34].

#### 3.4.2. The Effect of Fructose Concentration on Oxidative Stress

In addition to duration, fructose concentration significantly influences oxidative stress. As shown in Table 5, a dose‐dependent pattern emerged. Hepatic oxidative stress occurred even at lower fructose doses (10%), marked by declining antioxidant defenses, signaling an early onset of oxidative imbalance [16, 17, 33, 39, 41]. As intake increased to 30%–44%, the severity of oxidative stress became more pronounced, with elevated stress markers and reduced antioxidants [20, 21, 23, 26, 27, 30, 38, 40]. At high doses (60%–65%), oxidative damage became severe, overwhelming the liver’s defenses, increasing the liver’s susceptibility to damage [22, 29, 31, 32, 35, 36]. These findings suggest that even moderate fructose intake can trigger oxidative stress, which worsens with higher doses.

### 3.5. Metabolic and Histopathological Changes in Response to Fructose

Based on observation, not all categories were reported comprehensively among the 26 studies that documented secondary outcomes [16–41]. Furthermore, one study did not report on three secondary outcome categories, namely, weight, metabolic changes, and serum liver enzyme levels [19]. A total of seven studies omitted weight [19, 21, 23, 27, 35, 36, 41], one lacked metabolic [19], serum liver enzyme levels was absent in seven studies [19, 27, 30, 31, 33–35], and only four did not report histopathological changes [28, 32, 37, 38].

The results from 22 studies reporting histopathological changes showed that the most observed condition was fat/lipid accumulation in liver cells (13/26, 50%) [17, 21, 23, 25–27, 29, 30, 33, 36, 39–41], followed by steatosis (9/26, 34.6%) [17, 20–23, 33, 35, 36, 41]. When stratified by fructose concentration, the occurrence of steatosis appeared to be dose‐dependent, with steatosis reported in three studies using 60% fructose (3/26, 11.5%) [22, 35, 36], three studies using 30% fructose (3/26, 11.5%) [20, 21, 23], and three studies using 10% liquid fructose (3/26, 11.5%) [17, 33, 41], suggesting that both moderate and high fructose exposure may contribute to the development of hepatic steatosis. Changes in liver weight were also documented (7/26, 26.9%) [20–22, 24, 29, 34, 40]. These alterations were observed across a wide range of fructose concentrations (20%–60%), including one study using 20% [24], three studies using 30% [20, 21, 40], one study using 54% [34], and two studies using 60% fructose [22, 29], compared with studies using lower concentrations (10%), indicating a potential dose‐related pattern. Necrosis of hepatocytes was reported in five studies (5/26, 19.2%) [18, 23, 24, 27, 41], predominantly in experiments using moderate dose (10%–30%) with intervention durations ranging from 8 to 20 weeks. At lower fructose concentrations, the occurrence of necrosis appeared to be more dependent on the duration of exposure. Hepatocyte ballooning was observed in five studies (5/26, 19.2%) [20, 21, 36, 39, 40], primarily in studies that applied moderate to high fructose doses (10%–60%). Inflammation of liver cells was less frequently reported (3/26, 11.5%) [16, 23, 30]. Full details of histopathological outcomes are presented in Supporting Information 3, Table S2.

### 3.6. Quality Assessment of Included Studies

**FIGURE 2:**
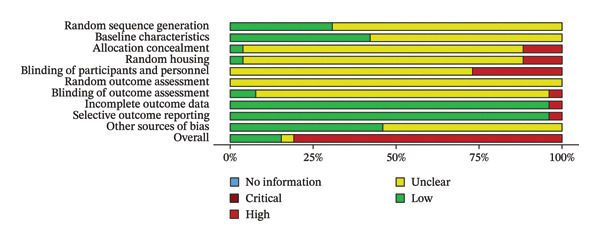
Summary plots of the risk of bias of the included studies.

In summary, the results show a significant variation in the quality of reporting across the studies, indicating a high proportion of unclear risk. This suggests that critical information regarding randomization, blinding, and handling of missing data was insufficiently reported, impacting the overall validity of the studies. The results of the assessment are illustrated in Figure 2 and Supporting Information 3, Figure S1.

## 4. Discussion

This systematic review summarizes the findings from 26 preclinical studies and establishes a consistent link between fructose consumption and the development of hepatic injury through oxidative stress. The primary markers of this fructose‐induced oxidative stress were identified as MDA, GSH, GSH‐Px, and SOD. A structured evaluation of the 26 included studies reveals consistent trends in redox disruption that depend on both the dose and duration of fructose intake. Our main findings from outcomes analysis are as follows: (i) Elevated oxidative stress biomarkers were observed across various doses and durations, confirming that fructose‐induced liver injury is a reproducible and dose‐responsive phenomenon [16–41]; (ii) prolonged fructose exposure impairs antioxidant defenses after an initial adaptive response; and (iii) mitochondrial dysfunction, endoplasmic reticulum (ER) stress, and enhanced lipogenesis act together to drive hepatic oxidative injury. The discussion highlights the biological pathways, time‐related progression, metabolic consequences, and the interplay between oxidative stress, lipogenesis, and ER stress in fructose‐induced hepatic injury.

### 4.1. Mechanistic Insights From Oxidative Stress Biomarkers

MDA was the most widely assessed marker of oxidative stress, reported in 14 of the 26 included studies [16–41]. Of these, a majority (11 studies) reported significantly elevated MDA levels following fructose exposure, reflecting sustained lipid peroxidation from chronic mitochondrial reactive oxygen species (ROS) production [16–41]. GSH and/or GSH‐Px were reported in 17 studies, with most demonstrating a significant reduction in hepatic GSH levels or GSH‐Px activity [18, 20, 25, 30, 32–34, 36–41]. This decline reflects the depletion of the GSH system, likely due to increased ROS burden, impaired nicotinamide adenine dinucleotide phosphate (NADPH) recycling, and mitochondrial damage. However, a minority of studies reported increased levels of GSH, GSH‐Px, or SOD. This suggests an early phase hepatic adaptation that can temporarily mitigate oxidative stress, but this response collapses with sustained exposure [17, 19, 22, 24, 35, 36].

Although oxidative stress emerged as a key mediator of fructose‐induced hepatic injury in this review, the relationship between fructose consumption and redox balance is increasingly recognized as context‐dependent. At lower fructose doses and shorter exposure durations, several studies reported the transient upregulation of antioxidant defenses, including increased GSH, SOD, and related enzymes, consistent with an early adaptive redox response rather than overt oxidative injury [17, 20, 43–45]. This biphasic response supports the concept that mild increases in ROS may serve as redox signaling cues that activate endogenous antioxidant systems and preserve hepatic redox homeostasis under moderate metabolic stress [45, 46].

Importantly, recent redox biology studies further support this interpretation by demonstrating that fructose‐related redox disturbances do not invariably result in overt hepatic oxidative injury, particularly at low to moderate, human‐relevant exposure levels [20, 46, 47]. Experimental evidence indicates that mild increases in ROS may activate redox‐sensitive signaling pathways and upregulate antioxidant defense systems, including GSH, SOD, and related enzymes, without inducing significant lipid peroxidation or structural liver damage, reflecting a physiological redox signaling response rather than pathological oxidative stress [17, 20, 43, 44]. Similar patterns have been observed in human studies, where fructose intake altered systemic redox states, particularly GSH/GSSG balance, without immediate structural liver injury, highlighting the dynamic and reversible nature of redox adaptation [48].

However, this adaptive capacity appears finite with increasing fructose dose or prolonged exposure. Antioxidant defenses become overwhelmed, leading to sustained redox imbalance, lipid peroxidation, mitochondrial dysfunction, and progressive hepatic injury [17, 44, 45]. Consistent with this framework, several studies included in the present review reported initial antioxidant upregulation at lower fructose doses, which subsequently declined with continued or higher‐dose exposure, supporting the transition from adaptive redox signaling to pathological oxidative stress once hepatic buffering capacity is exceeded. Together, these findings reinforce that oxidative stress is not merely a by‐product of fructose metabolism but a dose, time, and context‐dependent pathological driver of liver injury.

The consistent elevation of MDA indicates active lipid peroxidation, whereas the depletion of GSH, GSH‐Px, and SOD reflects the failure of cellular antioxidant defenses under metabolic overload. Together, these biomarker patterns illustrate how fructose‐induced redox imbalance progresses from early adaptive signaling to pathological oxidative stress, providing a mechanistic foundation for subsequent mitochondrial dysfunction and metabolic injury.

### 4.2. Mitochondrial Dysfunction Linked to Oxidative Stress

Compared to glucose, fructose is metabolized primarily in liver, bypassing key regulatory steps of glycolysis [49, 50]. This unregulated pathway promotes de novo lipogenesis, leading to fat accumulation, elevated triglycerides, and increased oxidative stress [9, 51]. A major mechanism connecting fructose metabolism to oxidative stress is mitochondrial dysfunction. Rapid conversion to triose phosphates, leads to an overproduction of acetyl‐CoA, overloading the electron transport chain and increasing ROS production through electron leakage [29, 34]. This metabolic pressure raises electron leakage and superoxide production, worsening the generation of ROS [52–56]. Mitochondrial impairment leads to adenosine triphosphate (ATP) deficiency, oxidative stress, and cellular damage [57]. Consistently elevated oxidative markers across studies confirm that ROS production is a persistent consequence of fructose metabolism [20, 21, 31, 36, 41].

The results from this systematic review show high levels of biomarkers related to the tricarboxylic acid (TCA) cycle process like acetyl‐CoA carboxylase (ACC) and ACC‐α, in response to fructose intake [29, 34]. Fructose is rapidly converted into triose phosphates glyceraldehyde‐3‐phosphate (G3P) and dihydroxyacetone phosphate (DHAP), leading to acetyl‐CoA overproduction, which enters the TCA cycle [58, 59]. Normally, acetyl‐CoA oxidation produces nicotinamide adenine dinucleotide (reduced form) (NADH) and flavin adenine dinucleotide (reduced form) (FADH_2_) to drive ATP synthesis via the ETC. However, excessive fructose saturates the TCA cycle, causing an overflow of reducing equivalents. This overload fuels the ETC and increases ROS production, especially at Complexes I and III, resulting in oxidative stress, cellular damage, and metabolic dysfunction [59]. Consistent with these findings, this systematic review shows persistent increases in lipid peroxidation biomarkers, such as MDA [16, 18, 22, 24, 27, 30, 33–35, 37, 39, 40], 4‐hydroxy‐2‐nonenal (4‐HNE) [28], thiobarbituric acid reactive substances (TBARSs) [21], and protein carbonyl (PC) content [20, 21], indicating sustained oxidative damage and confirming that fructose‐induced ROS production is a long‐term consequence of chronic exposure [44, 57, 60].

The result shows a strong correlation between the intervention duration and oxidative stress severity. Studies with short‐term (8–9 weeks) with 10%–20% fructose showed increased oxidative markers like MDA [24, 33, 37], while longer exposures (≥ 12 weeks) with higher doses (≥ 30%) consistently reported significant disruptions in MDA levels and redox depletions [27, 30, 34, 40]. Studies on prolonged exposure (≥ 16 weeks) to 30% and 54% fructose showed significant reductions in antioxidant defense mechanisms, including GSH depletion as well as decreased SOD and GSH‐Px activity [34]. However, some studies with exposure of 8–12 weeks at 10%, 20%, and 60% fructose showed compensatory upregulation of antioxidant enzymes in certain cases, such as increased GSH and SOD levels. This suggests that early phase hepatic adaptation can temporarily mitigate oxidative stress. However, extended fructose exposure exceeded the antioxidant system, leading to chronic liver injury [16–27, 29–31, 33–36, 39–41].

### 4.3. Crosstalk Between De Novo Lipogenesis (DNL) and Insulin Resistance–Induced Liver Injury

Fructose alone can drive metabolic and lipogenic changes without glucose coadministration. It is rapidly metabolized in the liver by ketohexokinase (KHK). The process bypasses essential glycolytic checkpoints, leading to uncontrolled substrate influx into DNL [46, 61]. This process is primarily mediated by the upregulation of sterol regulatory element–binding protein 1c (SREBP‐1c) and carbohydrate response element–binding protein (ChREBP). Studies from this systematic review have shown that without glucose, fructose consumption can increase the expression of SREBP‐1c, ChREBP, increasing enzymes like fatty acid synthase (FAS), ACC, and stearoyl‐CoA desaturase 1 (SCD1), which elevate hepatic triglyceride production and contribute to steatosis and dyslipidemia [22, 29, 34, 36, 38, 49, 54, 55, 62–65]. Furthermore, fructose inhibits the activity of peroxisome proliferator–activated receptor alpha (PPAR‐α), which is a key regulator of fatty acid oxidation. This shift in hepatic metabolism leads to lipid accumulation instead of oxidation [66]. Several studies showed that fructose independently increased the levels of liver X receptor alpha (LXR‐α) and PPAR gamma (PPAR‐γ), essential for both triglyceride synthesis and storage, along with increased very low–density lipoprotein (VLDL) secretion and microsomal triglyceride transfer protein (MTP), enhancing the assembly and export of VLDL particle levels. This mechanism led to hypertriglyceridemia and dyslipidemia observed in fructose‐fed models [17, 24, 25, 27, 33, 34, 49, 62, 63, 65, 67]. Another important factor in fructose‐induced dyslipidemia is the excessive production of retinol‐binding protein 4 (RBP4), which is associated with high hepatic insulin resistance and impaired lipid metabolism [34]. The upregulation of RBP4 mediated by fructose worsens systemic metabolic dysfunction by promoting lipogenic gene expression and decreasing insulin sensitivity [34, 66, 68]. These disruptions in insulin signaling are known to reduce hepatic glucose uptake and promote gluconeogenesis, creating a systemic state of insulin resistance. In line with this, our systematic review shows increases in insulin, glucose, and homeostatic model assessment for insulin resistance (HOMA‐IR), alongside decreases in AMP‐activated protein kinase (*p*‐AMPK/AMPK) and PPAR‐α levels, which are the hallmarks of hepatic insulin resistance and impaired β‐oxidation [18, 20, 21, 29–35, 37, 38, 40].

### 4.4. Inflammatory Pathways and Their Role in Redox Imbalance

Although not a primary endpoint of this review, inflammation consistently emerged as a critical mediator linking excessive fructose consumption to hepatic oxidative stress and disease progression. Our analysis found that several included studies demonstrated increased expression of these inflammatory mediators. Fructose can activate innate immune pathways like Toll‐like receptor and nuclear factor kappa‐light‐chain enhancer of activated B cells (NF–κB) as reported by three studies [27, 31, 34]. This activation upregulates proinflammatory cytokines including tumor necrosis factor‐α (TNF‐α) [16, 19, 20, 28–31, 34, 36, 39, 40], interleukin‐6 (IL‐6) [30, 31, 36, 39, 40], interleukin‐1β (IL‐1β) [28, 30, 31, 34, 36], and C‐reactive protein (CRP) [30, 35], which in turn impair insulin signaling and amplify mitochondrial dysfunction. These cytokines can form a vicious feedback loop by impairing mitochondrial membrane potential and increasing ROS production, further worsening the redox imbalance [20, 31, 36]. A key mechanism driving the progression of the hepatocellular injury is the activation of the NOD‐like receptor family pyrin domain containing 3 (NLRP3) inflammasome, which leads to cleavage of procaspase‐1 into its active form. This process ultimately results in the maturation of proinflammatory cytokines IL‐1β, IL‐18, fueling hepatocellular injury [69, 70]. In support of this, two studies in our review demonstrated that high fructose intake increased the hepatic expression of NLRP3 and its downstream effector, cleaved‐caspase‐1 [31, 36]. Interestingly, anti‐inflammatory interleukin‐10 (IL‐10) was found to be elevated in one study [34], suggesting a potential protective response. However, histological assessment in the same study revealed persistent inflammatory infiltrates, necrosis, and fibrosis, indicating that this IL‐10 increase was insufficient to halt disease progression. The coupling of elevated proinflammatory cytokines with oxidative stress markers such as MDA and TBARS supports the theory that inflammation is both a consequence and amplifier of hepatic oxidative injury [20, 30, 34, 36, 39, 40]. Together, these findings reinforce inflammation as a mechanistic bridge between metabolic dysfunction, redox imbalance, and hepatocellular injury.

### 4.5. ER Stress Genes Upregulated by Fructose Metabolism

Fructose‐induced oxidative stress is related to ER stress markers, hepatic lipid accumulation, and oxidative damage [20–22, 34, 36, 38]. Glucose‐regulated protein 78 (GRP78), a key marker of ER stress, is significantly elevated in liver fed with fructose, which triggers activation of the unfolded protein response (UPR). Consequently, this stress response leads to increased lipogenesis by activating p47 phox [34, 36]. ER stress and mitochondrial ROS production are also closely linked. This is because ER stress can increase mitochondrial ROS generation, while mitochondrial oxidative stress induces ER dysfunction, causing a harmful cycle of cellular damage [49, 52, 71, 72]. With chronic fructose exposure, ER stress activates activating transcription factor‐4 (ATF4), ATF‐6, and inositol‐requiring enzyme1‐α (IRE1‐α), which induce SREBP‐1c, leading to the upregulation of ACC and FAS and further promoting the DNL process and steatosis [22, 29, 34, 36, 49, 52, 54, 56, 73]. This sustained oxidative and inflammatory state increases the risk of hepatic metabolic disorders [5, 52, 74, 75].

### 4.6. Implications for Hepatic Metabolic Health: The Link to MAFLD

This study may have important implications for health professionals, policymakers, and other relevant stakeholders. Evidence shows that oxidative stress often precedes lipid accumulation, supporting its role as a key driver of liver injury. Generally, fructose‐induced lipid peroxidation activates hepatic stellate cells, initiating fibrotic changes that may lead to cirrhosis with long‐term exposure [5, 52, 75]. Importantly, the analysis indicates that fructose‐induced hepatic injury depends on both the dose and duration of exposure. At low to moderate fructose doses (10%–30%), longer exposure is required to cause noticeable liver damage [41]. In contrast, the highest‐dose fructose (65%) induced significant injury within 8 weeks [31]. These findings indicate that while prolonged exposure contributes to the persistence and progression of oxidative stress, the fructose concentration mainly determines the rate and severity of liver damage.

From a translational perspective, although fructose concentrations used in experimental models ranged from 10% to 66%, it is important to note that very high concentrations, particularly 65%, do not directly reflect typical daily human dietary intake. In human populations, fructose is primarily consumed through sucrose and HFCS, both of which contain substantial amounts of fructose, 50% in sucrose and ∼42%–55% in HFCS, often delivered in liquid form via sugar‐sweetened beverages [6, 7, 76–78]. Thus, high‐dose fructose models should be interpreted primarily as mechanistic tools rather than direct analogs of human consumption, whereas lower concentrations (10%–20%) may better approximate habitual exposure and early adaptive antioxidant responses, with higher doses accelerating oxidative damage and liver injury. Together, these models provide complementary insights into the spectrum of adaptive and pathological fructose‐induced hepatic dysfunction. In addition, health systems should focus on increasing awareness and educating the public about the harmful effects of chronic fructose intake, which can lead to continuous oxidative stress and promote hepatic fat and lipid accumulation [17, 21, 23, 25–27, 29, 30, 33, 36, 39–41], inflammation [16, 23, 30], necrosis [18, 23, 24, 27, 41], and fibrosis [31, 41].

### 4.7. Strengths and Limitations

This systematic review offers several key strengths that enhance its reliability. A major strength lies in the rigorous methodology, ensuring the inclusion of high‐quality preclinical studies through a structured selection process and evaluation using the SYRCLE risk‐of‐bias tool. Furthermore, a key advantage is the focused analysis on fructose alone, excluding coadministration with glucose or other diet, which allows clearer insights into its independent metabolic impact. The review also provides a comprehensive evaluation of oxidative stress and metabolic markers across varying fructose concentrations and durations. This dose‐ and time‐dependent analysis offers essential insights into the progression of hepatic dysfunction, particularly in the context of MAFLD. Additionally, it identifies research gaps and methodological inconsistencies, emphasizing the need for standardized study designs and more translational research to bridge animal and human studies.

Despite the comprehensive analysis of fructose‐induced hepatic oxidative stress and metabolic dysregulation, this systematic review has several limitations. First, because the search strategy focused on studies reporting hepatic oxidative stress to elucidate mechanistic links to fructose‐induced liver injury, responses at lower, human‐relevant fructose concentrations (10%–20%) may be underrepresented. At these doses, oxidative stress markers such as MDA were often elevated, but the magnitude and statistical significance of these changes varied, suggesting early or adaptive redox responses rather than overt oxidative injury. Studies reporting minimal or nonsignificant redox disturbances at lower fructose concentrations may therefore have been less likely to be captured, as such findings are not always highlighted in abstracts.

The time limitation might have excluded studies published before 2019. However, studies over the past 5 years have produced substantial and conclusive results. Another limitation is the significant sex bias in the included literature. Although sex was not an inclusion or exclusion criterion during study selection, 24 out of 26 included studies (92.3%) were conducted exclusively in male animals. This predominance likely reflects a long‐standing trend in preclinical research to use male models, often to avoid the hormonal variability in females (Cherubini et al., 2024). The lack of female representation in the preclinical data thus restricts our ability to extrapolate the observed effects of fructose to the broader population. This limitation underscores the urgent need for future studies to include both sexes more effectively, better reflecting the biological heterogeneity of liver disease. Finally, most included studies show a high or unclear risk of bias, particularly in randomization, blinding, and allocation concealment. Other limitations include lack of reporting; this lack of transparency is common in preclinical studies, where trials are often not explicitly labeled randomized controlled trials (RCTs), particularly when randomization is used. Consequently, many studies are rated as having unclear selection bias. This limits confidence in the results, specifically for subjective outcomes like histopathology, without clear blinding procedures. The limitations show the need for better adherence to reporting standards (Animal Research: Reporting of In Vivo Experiments [ARRIVE], SYRCLE) to strengthen future preclinical studies reliability and translational value.

## 5. Conclusion

In conclusion, this systematic review showed that excessive fructose consumption, without combination with another diet, could cause liver injury through oxidative stress, which then triggers the subsequent processes like inflammation. Overconsumption led to uncontrolled hepatic metabolism and overload. Furthermore, a persistent increase was observed in oxidative stress biomarkers. These biomarkers signified lipid peroxidation, protein oxidation, and oxidative damage, confirming that fructose‐induced ROS production was a long‐term effect of sustained exposure. Histopathological and biochemical changes showed the connection between excessive fructose intake and liver pathology. Fructose was found to drive DNL independently, increase hepatic triglyceride production, and promote VLDL secretion. This contributed to the accumulation of lipids in the hepatic cells that caused metabolic disease and fatty liver disease.

## Author Contributions

Marissa Arifin: conceptualization, data curation, formal analysis, methodology, validation, writing–original draft, writing–review and editing, and visualization. Wardatul Jannah: data curation, validation, formal analysis, and writing–review and editing. Neily Zakiyah: validation, formal analysis, and writing–review and editing. Anna Meiliana: conceptualization, methodology, validation, formal analysis, writing–review and editing, and supervision. Melisa Intan Barliana: conceptualization, methodology, writing–review and editing, and supervision. Keri Lestari: conceptualization, methodology, writing–review and editing, and supervision.

## Funding

The research did not receive funding from any sources.

## Conflicts of Interest

The authors declare no conflicts of interest.

## Supporting Information

Additional supporting information can be found online in the Supporting Information section.

## Supporting information


**Supporting Information 1** Supporting Information 1: PRISMA Checklist.

**Supporting Information 2** Supporting Information 2: PROSPERO registered and published document.

**Supporting Information 3** Supporting Information 3: Table S1: Search strategy, Table S2: Summary of study outcomes, and Figure S1: Traffic‐light plots reporting the risk of bias of the included studies.

## Data Availability

The data that support the findings of this study are available in the supporting information of this article.
