# Supplementary material for: Fructose Diet–Induced Liver Injury Through Oxidative Stress: A Systematic Review of Preclinical Studies
Source: J Nutr Metab. 2026 May 7;2026:1644860. doi: 10.1155/jnme/1644860 (PMC13150434; doi:10.1155/jnme/1644860)
Supplement: Supplementary file 2 — Supporting Information 2 Supporting Information 2: PROSPERO registered and published document. [file JNME-2026-1644860-s003.pdf]

NIHR | National Institute for Health and Care Research

PROSPERO
International prospective register of systematic reviews

Fructose Diet-Induced Oxidative Stress and Its Impact on Hepatic Oxidative Markers: A Systematic Review of Preclinical Studies

Marissa Arifin, Wardatul Jannah, Neily Zakiyah, Melisa Intan Barliana, Anna Meiliana, Keri Lestari

Citation
Marissa Arifin, Wardatul Jannah, Neily Zakiyah, Melisa Intan Barliana, Anna Meiliana, Keri Lestari. Fructose Diet-Induced Oxidative Stress and Its Impact on Hepatic Oxidative Markers: A Systematic Review of Preclinical Studies. PROSPERO 2025 CRD420251009741. Available from https://www.crd.york.ac.uk/PROSPERO/view/CRD420251009741.

REVIEW TITLE AND BASIC DETAILS

Review title
Fructose Diet-Induced Oxidative Stress and Its Impact on Hepatic Oxidative Markers: A Systematic Review of Preclinical Studies

Review objectives
This systematic review examines how excessive fructose consumption leads to hepatic oxidative stress in the liver and induced fat accumulation in hepatic cells

Context and rationale
Context:

Fructose has become a significant part of modern diets due to the widespread use of high-fructose corn syrup (HFCS) and added sugars in processed foods and beverages. While moderate fructose intake is generally well-tolerated, excessive consumption has increasingly been linked to metabolic disorders, mainly for metabolic-associated fatty liver disease (MAFLD). The liver is the primary site for fructose metabolism, where its rapid conversion into lipids can lead to excessive fat accumulation, oxidative stress, and subsequent liver dysfunction.

rationale:

Excessive fructose intake has been linked to hepatic oxidative stress and lipid accumulation, key contributors to metabolic diseases. Unlike glucose, insulin does not tightly control fructose

metabolism, resulting in increased lipogenesis and a greater vulnerability to oxidative damage. This oxidative stress arises from an overproduction of reactive oxygen species (ROS), mitochondrial dysfunction, and weakened antioxidant defenses in hepatic cells. Understanding the mechanisms behind fructose-induced hepatic oxidative stress and fat accumulation is essential for developing targeted interventions to address liver-related metabolic disorders. This systematic review aims to synthesize evidence on how excessive fructose consumption leads to hepatic oxidative stress in the liver, induced fat accumulation in hepatic cells, and impact on hepatic oxidative markers.

Keywords
Fructose diet; high fructose diet; hepatic oxidative stress; animal study

SEARCHING AND SCREENING

Searches
The literature was systematically searched using three databases: MEDLINE (PubMed), Scopus, and Web of Science.

Study design
Only randomized study types will be included.

Included
The selected population will focus exclusively on animal models limited to randomized controlled trials using a fructose diet as the intervention, exclusively without being combined with other diets. This review will include articles published in English over the next five years (2019–2024). A comparison of this systematic review will include a normal diet without fructose.

Excluded
This systematic review will exclude studies not conducted as in vivo animal studies, those without a control group, review articles, conference papers, and proceedings. Articles that cannot be accessed in full text after requesting access from the author will also be excluded.

Link to search strategy
A full search strategy has been uploaded to PROSPERO. The PDF may be accessed through this link https://www.crd.york.ac.uk/PROSPEROFILES/d0884b231d2bd95784d655cded77ca97.pdf.

ELIGIBILITY CRITERIA

Human disease modelled
metabolic-associated fatty liver disease (MAFLD)

Animals/Population

Included
The selected population focused exclusively on animal models (all species, all sexes)

Intervention(s) or exposure(s)

Included

The intervention inclusion criteria are fructose diet as the intervention, exclusively without being combined with other diets.

Comparator(s) or control(s)

Included
A comparator of this systematic review is a group of control with regular diet without fructose.

Other selection criteria or limitations applied

This review includes articles published in English over the past five years (2019–2024), published in English.

OUTCOMES TO BE ANALYSED

Outcome measure(s)

Included
Analyzing the impact of fructose-induced hepatic oxidative stress involved examining two outcomes. First, as primary outcome, hepatic oxidative stress was assessed using several biomarkers, including malondialdehyde (MDA), glutathione (GSH), glutathione peroxidase (GSH-Px), superoxide dismutase (SOD), and other related biomarkers of oxidative stress. Second, changes in weight, metabolic status, and liver histopathology were evaluated.

Excluded
Studies that do not have both outcomes will be excluded from this review

DATA COLLECTION PROCESS

Study selection and data extraction

Procedure for study selection
The search records from all databases will be exported to Rayyan.ai.new, a web-based intelligent systematic review application, and checked for duplicates. Screening processes will be conducted in two stages. The initial screening will be based on title and abstract, followed by full-text screening. Both stages will be reviewed independently by two reviewers. Any discrepancies will be resolved by consensus or discussions with a third and fourth reviewer.

Prioritise the exclusion criteria
This systematic review will exclude studies not conducted as in vivo animal studies, those without a control group, those without both outcomes, review articles, conference papers, and proceedings. Articles that cannot be accessed in full text after requesting access from the author will also be excluded.

Methods for data extraction
We will develop a data extraction sheet to collect essential characteristics and information for eligible studies. The sheet will be pilot-tested on five randomly selected analyses. One of the reviewers will conduct a thorough independent data extraction. Other reviewers will assess the data extraction process and clarify any uncertainties that arise.

Data to be extracted: study design
We will collect data related to characteristics of the study design: the author's name, year of publication, country of study, population species, sex, and sample size.

Data to be extracted: animal model
We will collect the population species and sex

Data to be extracted: intervention of interest
Furthermore, we will document details regarding the intervention strategy, including fructose concentration/dosage, follow-up duration

Data to be extracted: primary outcome(s)
We will collect the primary outcomes data all biomarkers related to hepatic oxidative stress: malondialdehyde (MDA), glutathione (GSH), glutathione peroxidase (GSH-Px), superoxide dismutase (SOD), and other related biomarkers of oxidative stress

Data to be extracted: secondary outcome(s)
We will collect the secondary outcomes data, all biomarkers related to changes in weight, metabolic status, and liver histopathology were evaluated.

Risk of bias and/or quality assessment
By use of SYRCLE's risk of bias tool

Method for risk of bias and/or quality assessment
Two reviewers will independently review the quality assessments. The quality of the included studies will be evaluated using the Systematic Review Centre for Laboratory Animal Experimentation (SYRCLE), which offers a Risk of Bias (RoB) Tool for animal intervention studies.Any discrepancies were resolved by consensus or discussions with a third and fourth reviewer.

PLANNED DATA SYNTHESIS

Strategy for data synthesis

Planned approach
For my systematic review, a narrative synthesis will be used to analyze and interpret the findings from included studies. This approach has been chosen due to anticipated heterogeneity in study designs, populations, interventions, and outcome measures, which may limit the feasibility of a meta-analysis.

Analysis of subgroups or subsets

Subgroup analyses
Subgroup analyses will be done for fructose concentration and duration of follow-up related to both outcomes

Sensitivity
Sensitivity to intervention

Given that different fructose diet interventions may have varying physiological and biological responses to the outcomes, I will perform sensitivity analyses by excluding the study that used combined diets with fructose.

Effect of Fructose Concentration

Sensitivity analyses will be performed to determine if variations in fructose concentration (e.g., 10%, 20%, 30%, etc.) alter the significance of observed on both outcomes

Stratification of Follow-Up Duration

To evaluate whether short-term vs. long-term fructose exposure influences hepatic oxidative stress differently, I will stratify studies based on follow-up duration and conduct a sensitivity analysis to assess whether findings remain consistent over time.

Handling of Missing Data

Since some studies may report incomplete outcomes, I will conduct a sensitivity analysis by excluding studies with missing data on primary or secondary outcomes to determine their impact on the overall synthesis.

Publication bias

Publication bias will be discussed narratively by assessing selective outcome reporting in the included studies (if applicable)

REVIEW AFFILIATION, FUNDING AND PEER REVIEW

Review team members

Mrs Marissa Arifin. Universitas Padjajaran Indonesia. Indonesia.

No conflict of interest declared.

Ms Wardatul Jannah. Universitas Padjajaran, Bandung, West Java. Indonesia.

No conflict of interest declared.

Dr Neily Zakiyah. Universitas Padjajaran, Bandung, West Java. Indonesia.

No conflict of interest declared.

Professor Melisa Intan Barliana. Universitas Padjajaran, Bandung, West Java. Indonesia.

No conflict of interest declared.

Dr Anna Meiliana. Universitas Padjajaran, Bandung, West Java. Indonesia.

No conflict of interest declared.

Professor Keri Lestari (review guarantor). Universitas Padjajaran, Bandung, West Java. Indonesia.

No conflict of interest declared.

Named contact

Mrs Marissa Arifin (marissa24001@mail.unpad.ac.id). Universitas Padjajaran Indonesia. Indonesia.

Review affiliation

Universitas Padjajaran, Bandung, West Java

Funding source

Review has no specific/external funding but is supported by guarantor/review team (non-commercial) institutions.

Additional information about funding

This study will support by a grant from the Center of Excellence in Higher Education for Pharmaceutical Care Innovation, Universitas Padjadjaran, Indonesia

TIMELINE OF THE REVIEW

Review timeline

Start date: 12 March 2025. End date: 12 June 2025.

Date of first submission to PROSPERO

12 March 2025

Date of registration in PROSPERO

12 March 2025

CURRENT REVIEW STAGE

Publication of review results

Results of the review will be published in English.

Stage of the review at this submission 1 change

Review stage	Started	Completed
Pilot work	✓	✓
Formal searching/study identification	✓	✓
Screening search results against inclusion criteria	✓	✓
Data extraction or receipt of IPD	✓	✓
Risk of bias/quality assessment	✓	✓
Data synthesis	✓	✓

Review status

The review is currently planned or ongoing.

ADDITIONAL INFORMATION

Review type

Pre-clinical animal intervention review

PROSPERO version history 1 change

- Version 1.1, published 12 Mar 2025
- Version 1.0, published 12 Mar 2025

Review conflict of interest

Declared individual interests are recorded under team member details.. No additional interests are recorded for this review.

Country

Indonesia

Medical Subject Headings

Fructose; Oxidative Stress

Revision note 1 change

update the review stages, review still start and on going

Disclaimer

The content of this record displays the information provided by the review team. PROSPERO does not peer review registration records or endorse their content.

PROSPERO accepts and posts the information provided in good faith; responsibility for record content rests with the review team. The guarantor for this record has affirmed that the information provided is truthful and that they understand that deliberate provision of inaccurate information may be construed as scientific misconduct.

PROSPERO does not accept any liability for the content provided in this record or for its use. Readers use the information provided in this record at their own risk.

Any enquiries about the record should be referred to the named review contact
