# Supplementary material for: Fructose Diet–Induced Liver Injury Through Oxidative Stress: A Systematic Review of Preclinical Studies
Source: J Nutr Metab. 2026 May 7;2026:1644860. doi: 10.1155/jnme/1644860 (PMC13150434; doi:10.1155/jnme/1644860)

**SUPPLEMENTARY MATERIAL 3**

**Table S1 –** Search strategy

| Database | Search Strategy |
| --- | --- |
| PubMed | ("Fructose"[MeSH Terms] OR "Fructose"[All Fields] OR "fructoses"[All Fields] OR "Fructose"[Title/Abstract] OR "high fructose diet"[All Fields] OR "high fructose diet"[Title/Abstract]) AND ("hepatic oxidative stress"[All Fields] OR "hepatic oxidative stress"[Title/Abstract]) |
| Scopus | ALL ( fructose ) OR TITLE-ABS ( fructose ) AND ALL ( "HIGH FRUCTOSE DIET" ) OR TITLE-ABS ( "HIGH FRUCTOSE DIET" ) AND ALL ( "HEPATIC OXIDATIVE STRESS" ) OR TITLE-ABS ( "HEPATIC OXIDATIVE STRESS" ) |
| Web of Science | ("Fructose"[MeSH Terms] OR "Fructose"[All Fields] OR "fructoses"[All Fields] OR "Fructose"[Title/Abstract] OR "high fructose diet"[All Fields] OR "high fructose diet"[Title/Abstract]) AND ("hepatic oxidative stress"[All Fields] OR "hepatic oxidative stress"[Title/Abstract]) |

**Table S2 –** Summary of study outcomes (n = 26)

| **No. of Study** | **Author** | **Concentration of Fructose (%)** | **Duration of Follow up (weeks)** | **Primary Outcome Compared to Control Group** | | | | **Secondary Outcome compared to control group** | | | |
| --- | --- | --- | --- | --- | --- | --- | --- | --- | --- | --- | --- |
|  |  |  |  | **Hepatic Oxidative Stress** | | | | **Weight Changes** | **Metabolic Changes** | **Liver Function** | **Histopathological Changes** |
|  |  |  |  | **MDA** | **GSH/GSH-Px** | **SOD** | **Other Biomarkers** |  |  |  |  |
| **Fructose 10%** | | | | | | | | | | | |
| 1 | Règgami et ll., 2021 | 10 | 9 | ⬆ (p<0.001) | ⬇ (GSH p<0.01, GSH-Px activity p<0.001) | N/A | ⬇ (GST, CAT p<0.001) | Not significant | ⬆ (glucose, insulin, HOMA-IR, QUICKI, TG, VLDL-C p<0.001; TC p<0.01; , LDL-C p<0.05, )  ⬇ (HDL-C p<0.001) | N/A | - micro- and macro-vesicular fatty infiltration - hepatic steatosis |
| 2 | Alemán, M. et al., 2019 | 10 | 12 | ⬆ (p<0.05) | ⬆ (GSH-Px activity, p<0.05) ⬇ (GSH level p<0.05) | ⬆ (p<0.05) | ⬆ (GST and CAT activity p<0.05) | ⬆ (p<0.05) | ⬆ (glocose, TG, TC, HDL-C, LDL-C, VLDL-C p<0.05) | ⬆ (AST, ALT p<0.05) | - Microvesicular steatosis - Lipid deposition in liver - Changes in gross mitochondrial morphology - Mitochondria appeared to be shorter and more round - TUNEL-positive hepatocyte |
| 3 | Yang Y et ll., 2019 | 10 | 12 | ⬆ (p≤0.05) | ⬇ (GSH-Px, p≤0.05) | ⬇ (p≤0.05) | ⬆ (Hepatic XO activity, p≤0.05) ⬇ (CAT, p≤0.05) | ⬆ (p≤0.05) | ⬆ (TG, UA p≤0.05) | ⬆ (AST, ALT)**ᴼ** | - The hepatocytes in some sections become bad distinct - Liver ballooning degeneration score - Fatty deposition in some Liver cells - Cells nucleus with pycnotic appearance |
| 4 | Abdelhamid Y et ll., 2021 | 10 | 16 | ⬆ (p=0.0206) | N/A | N/A | N/A | ⬆ (p<0.0001) | ⬆ (glucose, LDL-C p=0.0002; TG, leptin, adiponectine p<0.0001)  ⬇ (HDL-C p=0.0211) | ⬆ (AST, ALT p<0.0001) | - Severe congestion in central vein with inflammatory cells infiltration in the portal area mainly surrounding bile ducts |
| 5 | Zouaoui O et ll., 2021 | 10 | 20 | N/A | ⬇ (GSH) | ⬇ᴼ | ⬆ (ROS)ᴼ ⬇ (CAT)ᴼ | N/A | ⬆ (glucose, TG)ᴼ ⬇ (HDL-C)ᴼ | ⬆ (ALT, AST, ALK-P and TB)ᴼ | - Dilatation of portal space - Centrilobular vein - Sinusoids - Congestion of blood and massive necrosis of hepatocytes - Lymphocytic infiltration - Hepatic fat accumulation - Diffuse parenchyma steatosis - Foamy vacuoles associate with fibrosis |
| **Fructose 20%** | | | | | | | | | | | |
| 6 | Wang G et ll., 2020 | 20 | 8 | ⬆ (p<0.01) | ⬇ (GSH-Px activity p<0.01) | ⬇ (p<0.01) | N/A | ⬆ᴼ | ⬆ (glucose, insulin, HOMA-IR p<0.01) | ⬆ (AST, ALT p<0.01) | N/A |
| 7 | Gubur S et ll., 2022 | 20 | 8 | ⬆ᴼ | ⬆ (GSH)ᴼ | ⬆ᴼ | ⬆ (CAT)ᴼ | Not significant | ⬆ (VLDL, TG p<0.05) | ⬆ (AST, ALT p<0.05) | - The liver weight (P < 0.05) - Pycnotic nuclei and vacuolization in hepatocytes  - Endothelium ruptured of veins - Sinusoidal dilatation - Necrosis |
| 8 | Alim A et ll., 2023 | 20 | 10 | ⬆ (p<o.o5) | ⬇ (GSH)ᴼ | ⬇ᴼ | N/A | ⬆ᴼ | ⬆ (glucose, insulin, TC, TG, LDL-C, SFA, MUFAs, SCFA)ᴼ ⬇ (HDL-C)ᴼ | ⬆ (AST, ALT p<0.05) | - Cell vacuolization, congestion, - Single-cell necrosis with Suzuki scores of 10.20 |
| 9 | Altintas F et ll., 2022 | 20 | 16 | Not significant | N/A | ⬆ (p<0.05) | Not significant (CAT) | N/A | N/A | N/A | - Necrotic cells - vacuolated cells |
| 10 | Hernández et ll., 2020 | 20 | 18 | N/A | ⬇ (GSH-Px activity p<0.05) | ⬇ (p<0.05) | ⬇ (CAT and Total Antioxidant Activity, NO p<0.05; endothelin 1) | ⬆ (p<0.05) | ⬆ (TC, LDL-C, VLDL-C p<0.05) | ⬆ (AST, ALT p<0.05) | - exhibited cellular degeneration - massive fatty changes - cytoplasmic vacuolation - the loss of cellular boundaries |
| 11 | Mautone Gomes et ll., 2023 | 20 | 24 | N/A | N/A | N/A | Not significant (4-HNE, CML) ⬆ (NOS, p=0.0035; Total Thiolic content, p=0.0293) | Not significant | ⬆ (liver cholesterol, glocose) ⬇ (TG p=0.0015, fructosamine p=0.0262) | ⬆ (ALT p=0.0372) | N/A |
| **Fructose 30%** | | | | | | | | | | | |
| 12 | Bingül et ll., 2021 | 30 | 8 | N/A | N/A | N/A | ⬆ (ROS, TBARS, PC, DC, AOPP, serum AGE, hepatic AGE, CML p<0.05) | N/A | ⬆ (glucose, HOMA-IR, TG p<0.05)  Not significant (TC, Insulin) | ⬆ (AST, ALT p<0.05) | - Liver weight increase (p<0.05) - Steatosis (p<0.05) - Hepatocyte ballooning scores (p<0.05) |
| 13 | Bingul I et ll., 2021 | 30 | 8 | N/A | ⬇ (GSH-Px p<0.001) | ⬇ (p<0.01) | ⬆ (ROS, DC, PC, hepatic AGE, p<0.01; TBAR, Serum AGE, Hepatic AOPP, p<0.001; Serum CML p<0.05) | Not significant | ⬆ (HOMA IR, TG p<0.01) | ⬆ (AST p<0.01, ALT p<0.05) | - Liver weight increase - Microvesicular steatosis - Hepatocyte ballooning without fibrotic changes |
| 14 | EL Shial et ll., 2023 | 30 | 8 | N/A | N/A | N/A | ⬆ (iNOS, CYP2E1, c-JNK1 mRNA, p<0.05) | N/A | ⬆ (TC, LDL-C p<0.05) ⬇ (HDL-C p<0.05) | ⬆ (AST, ALT p<0.05) | - Inflammation around the central vein and hepatocytes with marked steatosis - Some cells exhibited necrosis - liver index (p<0.05) |
| 15 | Li C et ll., 2023 | 30 | 8 | N/A | ⬇ (GSH and GSH-Px activity, p<0.05) | ⬇ (p<0.05) | ⬆ (JNK/JNK, p-ERK/ERK, p38 MAPK, cleaved caspase-3, capase-3/cleaved caspase-3 ratios p≤0.0001; Anti Bax, Bax/Bcl-2 ratio*) | ⬆ᴼ | ⬆ (glucose, insulin, HOMA-IR p<0.05; TC, LDL-C, TG p<0.01) | ⬆ (AST, ALT p<0.05) | N/A |
| 16 | Iskender H et ll., 2022 | 30 | 12 | ⬆ (p<o.o02) | N/A | ⬇ (p<0.0001) | ⬆ (NF–κB, p<0.0001) | N/A | ⬆ (glucose, VLDL p<0.01; TG p<0.02)  ⬇ (HDL-C p<0.0001) | N/A | - Necrosis of the hepatocytes (p < 0.03) - Hyperplasia of the bile ducts (p < 0.05) - Fat droplets in the cytoplasm of hepatocytes - lymphocytes infiltrated intralocular and portal areas - Mallory–Denk bodies in the cytoplasm of some hepatocytes - Fatty acids in liver cells |
| 17 | Zhang X et ll., 2019 | 30 | 13 | ⬆ (p<0.05) | ⬇ (GSH-Px, p<0.05) | ⬇ (p<0.05) | N/A | ⬆ᴼ | ⬆ (TC, TG, Isulin p<0.05) ⬇ (HDL-C p<0.05) | ⬆ (AST, ALT, ALP p<0.05) | - Liver weight, liver index (p<0,05) - Excessive accumulation of lipid droplets inside the parenchyma cells - Ballooned lipid-laden hepatocytes - Severe cellular degeneration - Loss of cellular boundaries. |
| 18 | Quan X et ll., 2022 | 30 | 16 | ⬆ (p<0.01) | ⬇ (GSH-Px, p<0.01) | ⬇ (p<0.01) | N/A | ⬆ (p<0.05) | ⬆ (glucose, insulin, HOMA-IR, TC, TG, LDL-C, Apo-B p<0.01) ⬇ (HDL-C, Apo-A1 p<0.01) | N/A | - Liver index (p<0.01), fat index (p<0.05) - Enlarged numbers of lipid droplets and inflammatory cells |
| **Fructose ≥ 40% - 60%** | | | | | | | | | | | |
| 19 | Hsu Y et ll., 2021 | 44 | 8 | N/A | N/A | N/A | N/A | ⬆ (p=0.0036) | ⬆ (glucose, TG, LDL-C, leptin, adiponectine p<0.0001)  ⬇ (HDL-C p=0.0211) | ⬆ (AST, ALT p<0.0001) | - Hepatic tissue TG (P<0.0001)  - Hepatic tissue TC (P<0.0001)  - The distribution proportion and size of lipid droplets |
| 20 | Sakamuri A et ll., 2020 | 54 | 36 | ⬆ᴼ | ⬇ (GSH-Px)ᴼ | ⬇ᴼ | ⬆ (p47 *phox,* RBP4, ER stress gene, glucose regulated protein (GRP78), hepatic lipid peroxidation, ACCα, SCD1, SREBP1c,, PPAR-γ, LXR-α, ChREBP, PPAR-α)* | ⬆ᴼ | ⬆ (glucose, insulin, HOMA-IR, TC, TG, MCP-1)ᴼ | N/A | Liver tissue weights |
| 21 | Elseweidy M et ll., 2022 | 60 | 8 | ⬆ (p<0.001) | ⬆ (GSH, p<0.001) | N/A | ⬆ (ACL, aldolase B, FAS activities, SERBP-1c gene) ⬇ (p- AMPK, p<0.001, CTRP3 gene) | ⬆ (p<0.001) | ⬆ (UA)ᴼ | ⬆ (AST, ALT p<0.001) | - Liver weight (P<0.001) - steatosis - Necrotic with pyknotic nuclei - Cells infiltrations perivascularly and within portal areas - congested hepatic blood vessels - Interstitial lymphocytic infiltrations - Uric acid in iver tissue |
| 22 | Shawky NM et ll., 2019 | 60 | 9 | ⬆ (p<0.05) | ⬆ (GSH, p<0.001) | ⬇ (p<0.05) | N/A | N/A | ⬆ (glucose, insulin, HOMA-IR p<0.01, TC p<0.001, LDL-C p<0.05) ⬇ (HOMA-β index p<0.05) | N/A | - Semi-quantitative scores for hepatic steatosis (p<0.01) - Diffuse steatosis - The aortic vascular reactivity was greatly impaired |
| 23 | Park J et ll., 2020 | 60 | 10 | N/A | N/A | N/A | ⬆ (SREBP-1, FAS/b-actin, SCD-1/b-actin, Nrf2/b-actin, HO-1/b-actin) ⬇ (PPARα/b actin, p-AMPK/AMPK, p-ACC/ACC) | Not significant | ⬆ (glucose, insulin, HOMA-IR, TC, TG, LDL-C p<0.05)  ⬇ (HDL-C p<0.05) | ⬆ (AST, ALT p<0.05) | - liver weight increase (p<0.05) - accumulating larger lipid |
| 24 | Singh S et ll., 2023 | 60 | 10 | N/A | ⬇ (GSH, p<0.05) ⬆ (GSH-Px)ᴼ | ⬆ (p<0.05) | ⬆ (ROS, CAT, Grp78, Ire1α, Perk, Atf6, Atf4, eif2α, Ire1α (Ser-724), PERK (Thr-980), p-eIF2α (Ser-51), Txnip)ᴼ | N/A | ⬆ (glucose, TC, TG, UA)ᴼ | ⬆ (AST, ALT, ALP)ᴼ | - Hepatic steatosis and  - Ballooning of hepatocytes - TG in hepatic tissue - ChREBP SREBP-1c, SCD-1ACC, FAS in hepatic tissue |
| 25 | Rashwan A et ll., 2019 | 60 | 14 | N/A | ⬇ (GSH level p<0.05) | ⬇ (p<0.05) | N/A | ⬇ (p≤0.05) | ⬆ (glucose, HbA1c, HOMA-IR, TC, TG, LDL-C p<0.05)  ⬇ (HDL-C p<0.05) | ⬆ (AST, ALT p<0.05) | N/A |
| **Fructose > 60%** | | | | | | | | | | | |
| 26 | Rai R et ll., 2020 | 65 | 8 | N/A | N/A | Not significant | ⬆ (Catalase activity p≤0.05) Not significant (ROS activity, iNOS, NO level, Caspase 3 ) | Not significant | ⬆ (glucose, insulin, TG, UA p<0.05) | N/A | - indistinct and disarranged hepatocytes - liver fibrosis - massive deposition of the glycogen |

⬆: increase, ⬇: decrease, *: Significant with p ≤0.05, **: Not significant with p ≥0.05, X: not reported/not analyzed, ᴼ = did not provided with significant (p) value, 4-HNE: 4-Hydroxynonenal, 8–OHdG: 8-Hydroxy-2'-deoxyguanosine, ACC: Acetyl-CoA Carboxylase, ACC-α: Acetyl-CoA carboxylase-α, ACL: ATP citrate lyase, AGE: Advanced glycation end products, ALP: Alkaline Phosphatase, ALT: Alanine Aminotransferase, AMPK: AMP-activated protein kinase, AOPP: Advanced oxidized protein products, Apo-A1: Apolipoprotein-A1, Apo-B: Apolipoprotein B, ASC: Apoptosis associated speck like protein containing a CARD (caspase recruitment domain), AST: Aspartate Aminotransferase, ATF4: Activating Transcription Factor-4, ATF6: Activating Transcription Factor-6, CAT: Catalase, ChREBP: Carbohydrate response element-binding protein, CML: Carboxymethyl lysine, CRP: C-reactive protein, CTRP3: C1q/TNF-related protein 3, CYP2E1: Cytochrome P450 2E1, DC: Diene conjugates, eIF2-α: Eukaryotic translation initiation factor-α, ER: Endoplasmic Reticulum, ERK: Extracellular regulated kinase, FAS: Fatty acid synthase, GPX: Glutathione peroxidase, GRP78: Glucose-regulated protein, GSH: Glutathione, GSH-Px: Glutathione peroxidase, GST: Glutathione S transferase, HDL: High-Density Lipoprotein, HO-1: Heme Oxygenase-1, HOMA-IR: Homeostasis Model Assessment of Insulin Resistance, HOMA-β: Homeostasis Model Assessment –β, HSP-70: Heat Shock Protein -70, HYP: Hydroxyproline, IBA-1, Ionized calcium-binding adaptor molecule-1, IL: Interleukin, IL-1β: Interleukin -1β, IL-6: Interleukin-6, iNOS: Nitric Oxide Synthase Inducible, Ire1-α: Inositol-Requiring Enzyme1-α, JNK: c-Jun-N-terminal kinase, LDL: Low-Density Lipoprotein, LPC: Lyso phosphatidyl choline, LPE: Lyso phosphatidyl ethanolamine, LXR-α: Liver X Receptor Alpha, MCP1: Monocyte chemoattractant protein-1, MDA: Malondialdehyde, MPO: Myeloperoxidase, mRNA: Messenge ribonukleat, MUFAs: Monounsaturated Fatty Acids, NF–κB: Nuclear Factor kappa-light-chain-enhancer of activated B cells, NLRP3, NOD-like receptor family pyrin domain containing 3, NOS: Nitric Oxide Synthase, Nrf2: Nuclear factor erythroid-2, p-ACC: phosphorylated Acetyl-CoA Carboxylase, p-AMPK: phosphorylated AMP-activated protein kinase, PC: Protein carbonyl, p-eIF2α: Phosphorylated eukaryotic initiation-2α, PPAR-α: Peroxisome Proliferator-Activated Receptor Alpha, PPAR-γ: Peroxisome Proliferator-Activated Receptor Gamma, RANKL: Receptor Activator of Nuclear Factor-κB Ligand, RBP4: Retinol-binding protein, ROS: Reactive oxygen species, SCD: Stearoyl CoA desaturase, SCD-1: Stearoyl-CoA desaturase-1, SCFA: Short-Chain Fatty Acids, Ser-51: Serine-51, Ser-724: Serine-724, SFA: Saturated Fatty Acids, SOD: Superoxide dismutase, SREBP-1: Expression of sterol regulatory element-binding protein 1, SREBP1c: Expression of sterol regulatory element-binding protein 1c, TB: Total Bilirubin, TBAR: Thiobarbituric acid reactive substances, TC: Total Cholesterol, TG: Triglycerides, Thr-980: Threonine-980, TLR2: Toll-like receptor 2, TLR4: Toll-like receptor 4, TNF-α: Tumor Necrosis Factor-α, TNF-β: Tumor Necrosis Factor-β, Trx: Thioredoxin, Txnip: Thioredoxin-interacting protein, UA: Uric Acid, VLDL: Very Low-Density Lipoprotein.

**Figure S1 –** Traffic-light plots reporting the risk of bias of the included studies.


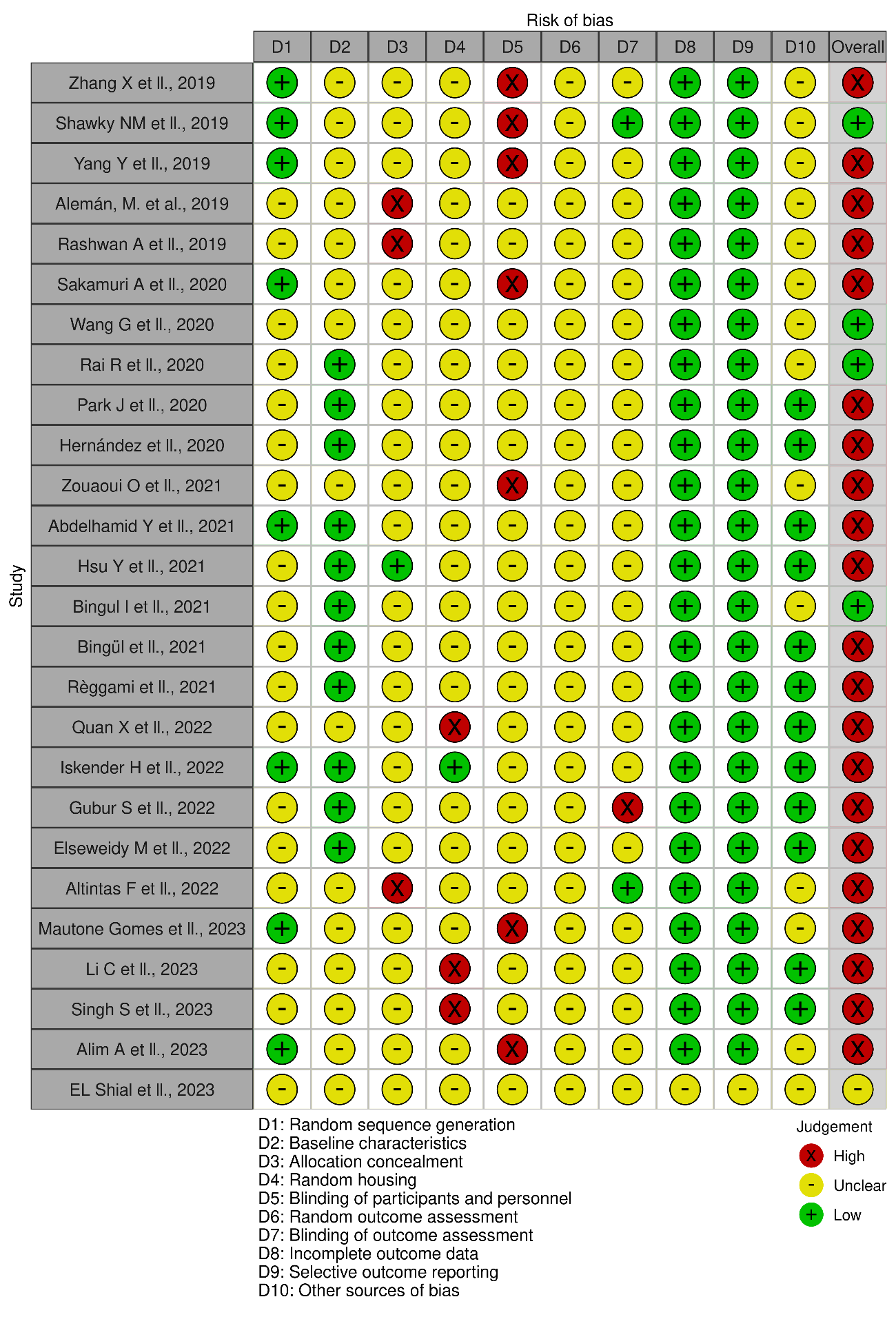

Supplement: Supplementary file 3 — Supporting Information 3 Supporting Information 3: Table S1: Search strategy, Table S2: Summary of study outcomes, and Figure S1: Traffic‐light plots reporting the risk of bias of the included studies. [file JNME-2026-1644860-s001.docx]
